# Azithromycin Mitigates Experimental Cryptosporidiosis-Driven Ileocecal Adenocarcinoma by Modulating Autophagy, Apoptosis, and PI3K/AKT Signaling

**DOI:** 10.3390/biomedicines14061232

**Published:** 2026-05-29

**Authors:** Walaa H. El-Maadawy, Eman S. El-Wakil, Marwa Hassan, Gamal A. Abo Sheishaa, Noha F. Zahran, Mohammed S. El Faramawy, Mohammed H. Abdallah, Eman A. Elsayed

**Affiliations:** 1 Pharmacology Department, Theodor Bilharz Research Institute, Giza 12411, Egypt; 2 Department of Parasitology, Theodor Bilharz Research Institute, Giza 12411, Egypt; 3 Immunology Department, Theodor Bilharz Research Institute, Giza 12411, Egypt; 4 Medical Parasitology Department, Faculty of Medicine, Al-Azhar University, Cairo 11884, Egypt; 5 Medical Parasitology Department, Faculty of Medicine, Al-Azhar University, New Dameitta 34517, Egypt

**Keywords:** apoptosis, autophagy, azithromycin, *Cryptosporidium parvum*, drug repurposing, ileocecal adenocarcinoma, nitazoxanide, PI3K/Akt signaling, TRAIL pathway

## Abstract

**Background/Objectives:** *Cryptosporidium parvum* (*C. parvum*), a waterborne intestinal parasite, causes severe, persistent infections in immunocompromised hosts and has been linked to the onset of ileocecal adenocarcinoma. However, the molecular pathways linking chronic infection to carcinogenesis remain unclear. Nitazoxanide (NTZ), the only FDA-approved drug for this infection, shows limited efficacy. In contrast, azithromycin (AZM) possesses both antiparasitic and anticancer activity, though conclusive evidence supporting its effectiveness against cryptosporidiosis is still lacking. This study aimed to investigate the therapeutic potential of AZM against chronic cryptosporidiosis and its associated tumorigenic sequelae. **Methods:** Immunosuppressed mice were infected with *C. parvum* and treated with NTZ or AZM. Parasite burden was assessed by quantifying fecal oocyst shedding. Ileocecal tissues were analyzed for histopathology, inflammation (IL-6 and TNF-α), autophagy markers (LC3II, Beclin-1, and Atg7), PI3K/AKT signaling, and apoptotic markers (Bcl2, Bax, cleaved caspase-3, DR4, and DR5) using ELISA, real-time PCR, and Western blot. **Results:** Chronic *C. parvum* infection induced Vienna 4.4 adenocarcinoma, activated autophagy and PI3K/AKT signaling, and suppressed intrinsic and TRAIL-mediated apoptosis. AZM significantly reduced the parasitic load by 87%, outperforming NTZ (62%). It also restored epithelial integrity, attenuated inflammation, and counteracted pro-tumorigenic effects by inhibiting autophagy, downregulating the PI3K/AKT pathway, and stimulating apoptosis. **Conclusions:** AZM counteracted parasite-driven tumorigenic mechanisms by disrupting survival pathways and promoting apoptosis in infected and transformed cells. These findings provide evidence that AZM exerts dual antiparasitic effects and counteracts pro-tumorigenic signaling in chronic cryptosporidiosis, highlighting its potential as a therapeutic agent to prevent infection-associated ileocecal carcinogenesis.

## 1. Introduction

*Cryptosporidium* causes global waterborne diarrhea outbreaks affecting both immunocompetent and immunosuppressed individuals [1], with a global prevalence of 7.2% [2]. The severity of the disease depends mainly on the host’s immune status. In immunocompetent hosts, infection usually causes self-limited watery diarrhea within approximately two weeks [3]. On the contrary, immunocompromised patients, including those with acquired immune deficiency syndrome (AIDS), on cancer chemotherapy, or organ transplant recipients, as well as pregnant females, children, and infants, are more liable to persistent, life-threatening conditions with a notification rate of 1.8 per 100,000 population in the EU/EEA [4]. The parasite also accounts for 10–15% of severe diarrheal illness in middle and low-income countries [5]. Symptoms include chronic diarrhea, water and electrolyte imbalances, malnutrition, and cognitive deficits in young children [6,7,8]. In addition, persistent infections are linked to several cancers, including hematological malignancies, colorectal cancer, and liver cancer. In particular, mounting evidence links *Cryptosporidium* spp. infections, notably those caused by *C. parvum*, with gastrointestinal cancers such as colon cancer and ileocecal adenocarcinoma [9,10,11,12], with prevalence ranging from 12.6% to 21% in individuals with molecularly confirmed infections [13]. Experimental studies have further substantiated the tumorigenic potential of *C. parvum*, demonstrating its ability to induce ileocecal and colorectal adenocarcinomas in animal models [3,14,15,16]. These studies have allowed detailed exploration of host–parasite interactions, immune responses, and early oncogenic events in cryptosporidiosis, thereby offering mechanistic insights that complement findings from clinical observations.

Cryptosporidiosis is a fecal–oral transmitted disease [17,18]. Infection begins after ingestion of the oocyst stage, which transforms into motile sporozoites. The sporozoites are released into the intestinal lumen, where they invade and attach to the apical surface of intestinal epithelial cells (IECs). The parasite then undergoes three cycles of asexual replication before transitioning to the sexual phase. During this phase, fertilization occurs, producing new infectious oocysts that can either be excreted in the feces to infect new hosts or remain in the intestinal lumen to reinfect the same host [19].

Like other carcinogenic pathogens, *C. parvum* infection disrupts the host’s cellular processes involved in survival, proliferation, autophagy, and immune evasion [20]. *C. parvum* also interferes with various host signaling pathways that support its survival and promote cancer progression, including the phosphoinositide 3-kinase (PI3K)/protein kinase B (Akt) signaling pathway [21]. Despite the global burden of cryptosporidiosis with its evidenced tumorigenic potential, therapeutic options remain limited to supportive therapy and nitazoxanide (NTZ), the first-line drug. However, NTZ exhibits limited impact in malnourished children and is inadequate in treating immunocompromised hosts [22].

Interestingly, pharmacological studies demonstrated the effectiveness of repositioning antimicrobial drugs in cancer therapy [1,23]. Azithromycin (AZM), a macrolide antibiotic, inhibits bacterial protein synthesis by binding to the 50S subunit of the bacterial ribosome [24]. Compared to other macrolides, AZM displays a broader spectrum of activity, more favorable pharmacodynamics, an extended half-life due to its accumulation within lysosomes in phagocytic cells, and its slow release into the bloodstream, thus providing adequate therapeutic coverage for several days’ post-completion of treatment [25]. It is also effective at lower doses, has fewer side effects, and offers the potential for long-term administration [26].

In addition to its antimicrobial activities, AZM has shown potent immunomodulatory effects by reducing neutrophil and eosinophil functions and lowering interleukin (IL)-8, IL-1beta, IL-6, and tumor necrosis factor-alpha (TNF-α) levels [27]. Furthermore, AZM exhibits robust anticancer effects, including against colon cancer, by inducing the tumor necrosis factor-related apoptosis-inducing ligand (TRAIL). This induces upregulation of the death receptors DR4 and DR5, leading to autophagy inhibition and selective targeting of tumor cells [28]. AZM also inhibits angiogenesis and tumor cell proliferation by downregulating PI3K/Akt signaling [29].

The antiparasitic activities of AZM were demonstrated in several studies against malaria [30], *Toxoplasma gondii* [31,32,33], and *Giardia intestinalis* [34]. However, its parasitological efficacy in treating human cryptosporidiosis remains debatable. Several case reports have documented the effectiveness of AZM in treating cryptosporidiosis in both immunocompetent and immunocompromised children, resulting in reduced parasite burden and alleviated clinical symptoms [35,36,37,38]. Yet, earlier studies have reported limited parasitological efficiency [39]. Dionisio et al. [40] have proposed that short-term treatment with AZM was ineffective in achieving complete parasitological [1] elimination or preventing relapses in HIV-infected individuals. In contrast, prolonged maintenance therapy at low doses exhibited marked clinical and parasitological improvements.

Despite these controversies, this study aimed to elucidate the molecular mechanisms by which chronic *C. parvum* infection drives ileocecal adenocarcinoma, focusing on autophagy, apoptosis, and PI3K/AKT signaling. We also compared the therapeutic efficacy of AZM against cryptosporidiosis with that of the standard drug, NTZ. In addition, we assessed the potential of AZM to *C. parvum*-induced tumorigenesis by modulating host inflammatory responses, autophagic flux, apoptotic pathways, and oncogenic signaling.

## 2. Materials and Methods

### 2.1. Animals

Swiss albino mice (male, *n* = 40, weight ranges from 25 to 28 g) were housed at the animal house of Theodor Bilharz Research Institute (TBRI). The Ethics Committee at the Faculty of Medicine for Boys, Al-Azhar University, Egypt, approved the study protocol (PT: #0038, 11 February 2024), and all experiments adhered to the ARRIVE guidelines. Mice were kept in polypropylene cages under controlled conditions (22–24 °C and a 12 h dark/light cycle) with ad libitum access to food and water.

### 2.2. Induction of Immunosuppression and C. parvum Infection

Mice were orally administered dexamethasone (DEXA, Dexamethasone^®^, Amriya Pharmaceutical Ind., Alexandria, Egypt) in a dose of 0.25 mg/kg daily for two successive weeks, before being inoculated with *Cryptosporidium* spp. oocysts. The immunosuppressed mice continued to receive the same dose of DEXA throughout the experiment [41].

The Lumb’s technique was applied to isolate *Cryptosporidium* spp. oocysts from diarrheic calves [42]. Genetically identified *C. parvum* oocysts [43] were preserved in 2.5% potassium dichromate (K_2_Cr_2_O_7_) at a 1:1 ratio and kept at 4 °C until before animal inoculation [44]. The oocysts were rinsed at least 3 times with distilled water to remove traces of K_2_Cr_2_O_7_, then centrifuged at 1500× *g* for 15 min until a clear pellet was obtained [45]. Each mouse received about 10^4^ *C. parvum* oocysts [46], except for groups I and II, which were designated as 0 weeks post-infection (wpi).

### 2.3. Animal Groups

Mice were randomly allocated into five animal groups (*n* = 8/group), as follows: Group I (DEXA): normal mice received DEXA; Group II (DEXA + AZM): DEXA-treated mice were administered 100 mg/kg AZM [47] (Zithromax^®^, Pfizer, S.A.E., Cairo, Egypt) via oral gavage once per day for 2 weeks starting from the beginning of 9 wpi concurrently with DEXA; Group III (DEXA + *C. parvum*): DEXA-treated mice were infected with oocysts; Groups IV (DEXA + *C. parvum* + NTZ) and V (DEXA + *C. parvum* + AZM): infected and immunocompromised mice were administered NTZ (Nitazoxanide^®^, Utopia, S.A.E., Sharkia, Egypt) and AZM at doses of 200 mg/kg [14,41], and 100 mg/kg, respectively, via oral gavage once per day for 2 weeks starting from 9 wpi.

Twenty-four hours’ post-treatment, mice were humanely euthanized via inhalation of light isoflurane. Next, intestinal tissues were excised and processed for succeeding enzyme-linked immunosorbent assay (ELISA), real-time polymerase chain reaction (PCR), Western blot, and histopathological investigations.

### 2.4. Parasitological Analysis

Fecal pellets were collected weekly, starting from week 1 until the end of the experiment (week 10). Fecal samples were collected from each mouse, and the oocyst count was determined using cold Kinyoun’s acid-fast stain (Sigma-Aldrich, St. Louis, MI, USA), where the pellets were gathered from each mouse, and the average count of oocysts per group was determined. The fecal material (1 mg) was stored in one mL of formalin (10%). The resulting suspension was centrifuged at 500× *g* for 10 min, and a 100 µL aliquot of the concentrate was dried, stained, and examined using an oil lens (×100 magnification). The oocyst count per fecal sample (1 mL) was determined by calculating the average of three counts and multiplying by 10 [48], and the final oocyst number was expressed per gram of feces [49,50]. Additionally, the reduction in the percentage (PR%) of oocyst shedding was quantified as follows [51]:

PR%=I−T/I∗100

I: group infected with *C. parvum* and T: group that received treatment.

### 2.5. Histopathological Examinations

Ileum and ileocecal segments were fixed in formalin (10%) and embedded in paraffin wax. Sections of five µm thickness were stained with hematoxylin and eosin (H&E, Sigma-Aldrich, USA).

Inflammatory and degenerative changes were graded semi-quantitatively using the following criteria:

- Crypt epithelium and surface degeneration: Normal morphology = 0; Mild degeneration in surface and crypt epithelium = 1; Moderate degeneration = 2; Severe degeneration/necrosis = 3
- Villus degeneration: Intact villus structure = 0; Mild shortening/blunting = 1; Moderate villus atrophy = 2; Severe villus destruction = 3
- Infiltration of inflammatory cells: Absence of pathological infiltration = 0; Mild focal infiltration = 1; Moderate multifocal infiltration = 2; Severe diffuse inflammation = 3.

For neoplastic changes, the ileocecal tissue alterations were classified based on the Vienna classification of epithelial gastrointestinal neoplasia [52,53].

### 2.6. Enzyme-Linked Immunosorbent Assay (ELISA)

Ileocecal tissues were subjected to homogenization in PBS (1:10 *w*/*v*) and centrifuged (10,000× *g*, 4 °C) for 10 min. The supernatants were then used to quantify IL-6, TNF-α, Bcl2, Bax, and cleaved caspase-3 levels using the commercially available ELISA (Abbexa Ltd., Cambridge, UK). The BCA protein assay kit (Intron Biotechnology, Seongnam-si, Republic of Korea) was used to determine the total protein content of each sample.

### 2.7. Quantitative Real-Time PCR

Total RNA was isolated from ileocecal tissue utilizing an RNA extraction kit, and reverse-transcribed into cDNA according to the manufacturer’s instructions (Intron Biotechnology, Seongnam-si, Republic of Korea). Real-time PCR was performed using the Maxima SYBR Green master mix (Intron Biotechnology, Seongnam-si, Republic of Korea) with StepOne Software v2.3 (Applied Biosystems, Carlsbad, CA, USA). The expression of DR4, DR5, LC3II, Atg7, and Beclin-1 genes (Table 1) was analyzed, and GAPDH was used for normalization. Relative quantification was determined using the 2^−ΔΔCt^ method [54].

### 2.8. Western Blotting

Ileocecal tissues were lysed in the buffer of the Radio Immunoprecipitation Assay (RIPA), and total protein was isolated via the ReadyPrep^TM^ kit. Protein concentrations were determined following the Bradford Assay Kit protocol. Aliquots of 20 μg protein samples were combined with Laemmli buffer (2x) (pH = 6.8). The mixture was then loaded onto a polyacrylamide gel for electrophoresis (SDS-PAGE) and transferred to a polyvinylidene difluoride (PVDF) membrane. The membrane was incubated with the primary antibodies at 4 °C overnight, diluted in TBST (1:5000), including anti-PI3K (#sc-1637, Santa Cruz Biotechnology, Dallas, TX, USA), anti-phosphorylated (p)PI3K (#17366, Cell Signaling Technology, Sacramento, CA, USA), anti-AKT (#9272, Cell Signaling Technology, Sacramento, CA, USA), and anti-pAKT (#sc-293125, Santa Cruz Biotechnology, Dallas, TX, USA). The membrane was then incubated for 1 h with a horseradish peroxidase (HRP)-conjugated goat anti-rabbit secondary antibody (#7074, Cell Signaling Technology, Sacramento, CA, USA) at room temperature. The protein bands were captured using a CCD camera-based imager (Bio-Rad, Hercules, CA, USA) and their band intensities were quantified with ChemiDoc MP image analysis software (v3.0.1, USA), normalized to β-actin as a loading control.

### 2.9. Statistical Analysis

The data are expressed as the mean ± SD. Statistical comparisons and graph generation were performed using GraphPad Prism software (v9.0.0, USA). The normality of data was assessed before conducting statistical analyses using the Shapiro–Wilk test. Parametric data were analyzed by the one-way analysis of variance (ANOVA) followed by Tukey’s post hoc test. In contrast, nonparametric data were analyzed using the Kruskal–Wallis test, followed by Dunn’s post hoc test for multiple comparisons. A *p*-value of less than 0.05 was considered statistically significant.

## 3. Results

### 3.1. AZM Suppressed the Shedding of C. parvum Oocysts

**Figure 1:**
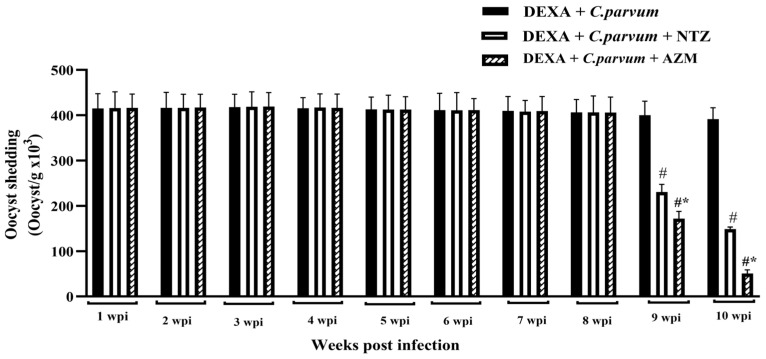
Oocyst shedding in different study groups. Results are expressed as mean ± SD. # *p* < 0.05 vs. *C. parvum*-infected group, * *p* < 0.05 vs. NTZ-treated group.

**Figure 2:**
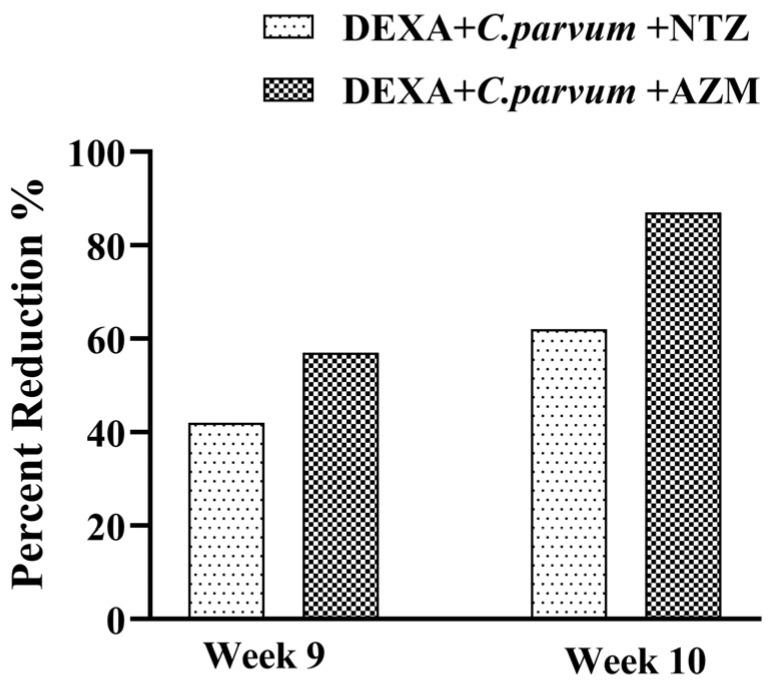
The percentage of *C. parvum* oocyst reduction for each study group in different weeks post-treatment.

Oocyst shedding was monitored weekly across all study groups from infection till the end of the study (10 wpi). No significant differences were observed between groups before drug administration (1 to 8 wpi). One-week post-treatment (9 wpi), both groups treated with NTZ or AZM significantly reduced oocyst counts (42% and 57%, respectively) compared to the group infected with *C. parvum*. By the end of 10th wpi, the AZM-treated group demonstrated superior efficacy, achieving an 87% reduction in oocyst shedding, which was significantly greater than the 62% reduction observed in the NTZ-treated group (Figure 1 and Figure 2).

### 3.2. Azithromycin Improved the Histopathological Alterations and Ileocecal Neoplasia Driven by Chronic Cryptosporidiosis

Histological analysis of ileum and ileocecal sections from DEXA-treated or DEXA + AZM-treated mice revealed preserved mucosal architecture, with intact finger-like villi lined by tall columnar epithelial cells and scattered goblet cells. Crypts and glands were uniformly arranged, showing minimal collagen accumulation and no signs of inflammation or hemorrhage.

In contrast, *C. parvum*-infected tissues exhibited severe mucosal injury, including extensive villus damage, epithelial loss, sub-epithelial edema, goblet cell depletion, and crypt dilation with epithelial desquamation. Dense mononuclear inflammatory infiltrates (lymphocytes, macrophages, and occasional eosinophils) were present. Parasites were frequently detected, contributing to architectural disruption.

NTZ treatment led to mild improvement, with short, thin villi, epithelial shedding at the tips, reduced goblet cells, persistent crypt dilation, and mild inflammatory infiltrates. Parasites remained detectable. AZM treatment showed notable mucosal recovery, with mild epithelial shedding and increased goblet cell presence. Inflammatory infiltrates were mild.

**Figure 3:**
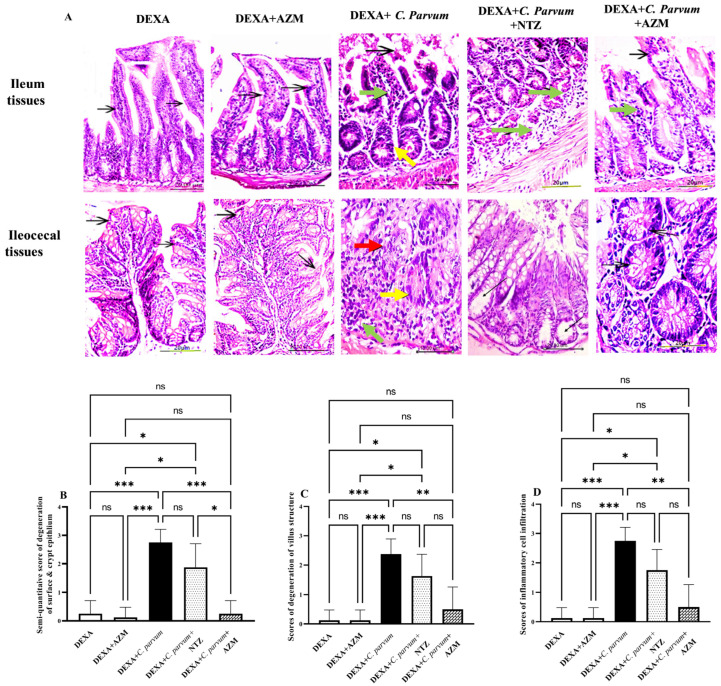
AZM attenuated the *C. parvum*-induced intestinal injury and ileocecal adenocarcinoma. (**A**) Photomicrographs of ileal mucosal tissues (upper panel) from groups treated with DEXA- and DEXA + AZM (black arrows: normal intact epithelial lining arrangement), DEXA + *C. parvum* (green arrows: inflammatory infiltrate with overlying disrupted surface epithelial lining. The yellow arrow: high-grade dysplastic cellular changes with respected glandular basement membrane and underlying muscularis mucosa); DEXA + *C. parvum* + NTZ and DEXA + *C. parvum* + AZM (black arrows: regenerative changes and green arrows: inflammatory infiltrate). Moreover, the lower panel showed ileocecal mucosal photomicrographs of the groups given either DEXA or DEXA + AZM (black arrows: normal surface epithelial lining alignment); *C. parvum* infection (green arrows: inflammatory infiltrate. The yellow arrow: high-grade dysplastic cellular changes. the red arrow: distortion of glandular architecture and glandular basement membrane yet still respecting underlying muscularis mucosa); DEXA + *C. parvum* + NTZ-treated group (black arrows: glandular cystic dilatation); and DEXA + *C. parvum* + AZM (black arrows: hyperactive glandular goblet cells and intact epithelial lining). Semi-quantitative scores for (**B**) crypt and surface epithelium degeneration, (**C**) villus degeneration, and (**D**) inflammatory cells infiltration. Data are displayed as mean ± SD, * *p* < 0.05, ** *p* < 0.01, *** *p* < 0.001, ns: non-significant. DEXA: dexamethasone; NTZ: nitazoxanide; AZM: azithromycin. Scale bars: 20 µm.

In the ileocecal region, *C. parvum* infection caused marked epithelial desquamation, glandular disruption, cellular atypia, and nuclear pleomorphism. Among eight samples, five showed intramucosal carcinoma (Vienna category 4.4), and three had low-grade mucosal neoplasia (Vienna category 3). Parasite accumulation, goblet cell depletion, vascular dilation, and dysplastic changes were prominent. NTZ-treated ileocecal tissues showed partial improvement, with five samples displaying low-grade dysplasia (Vienna category 3), and three with non-invasive carcinoma (Vienna category 4.2). Parasites adhered to absorptive cells, and leukocyte infiltration was extensive. AZM treatment reduced epithelial sloughing and revealed cystic gland dilatation with indefinite dysplasia (category 2) in three samples (Figure 3).

### 3.3. Azithromycin Suppressed the Inflammatory Responses in Ileocecal Neoplasia Driven by Chronic Cryptosporidiosis

**Figure 4:**
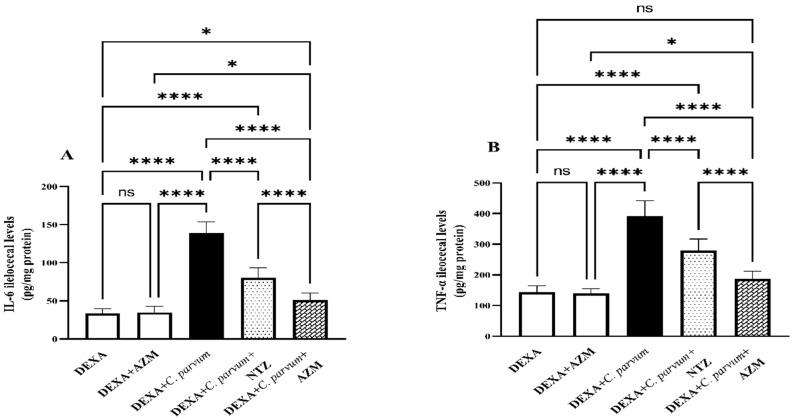
AZM mitigated the inflammatory reactions in *C. parvum*-driven ileocecal adenocarcinoma. Levels of (**A**) IL-6 and (**B**) TNF-α in ileocecal tissues. Data are displayed as mean (*n* = 8) ± SD, * *p* < 0.05, **** *p* < 0.0001, ns: non-significant. DEXA: dexamethasone; NTZ: nitazoxanide; AZM: azithromycin.

*Cryptosporidium* infection exacerbated the pro-inflammatory responses in immunosuppressed mice, as manifested by 4.14- and 2.72-fold increases in the level of pro-inflammatory cytokines “IL-6 and TNF-α” in ileocecal tissues, respectively, relative to DEXA-administered mice. Additionally, NTZ caused a partial reduction in IL-6 and TNF-α compared with mice infected with *C. parvum*, though their levels remained significantly elevated relative to the DEXA-treated mice. AZM markedly reduced TNF-α and IL-6 levels relative to mice infected with *C. parvum* or treated with NTZ, suggesting that AZM effectively counteracts *Cryptosporidium*-induced inflammation. Yet, the AZM-treated group showed a minor elevation in cytokine levels compared with the DEXA + AZM-treated group, as presented in Figure 4.

### 3.4. Azithromycin Inhibited the Induced Autophagy in Ileocecal Adenocarcinoma Driven by Chronic Cryptosporidiosis

**Figure 5:**
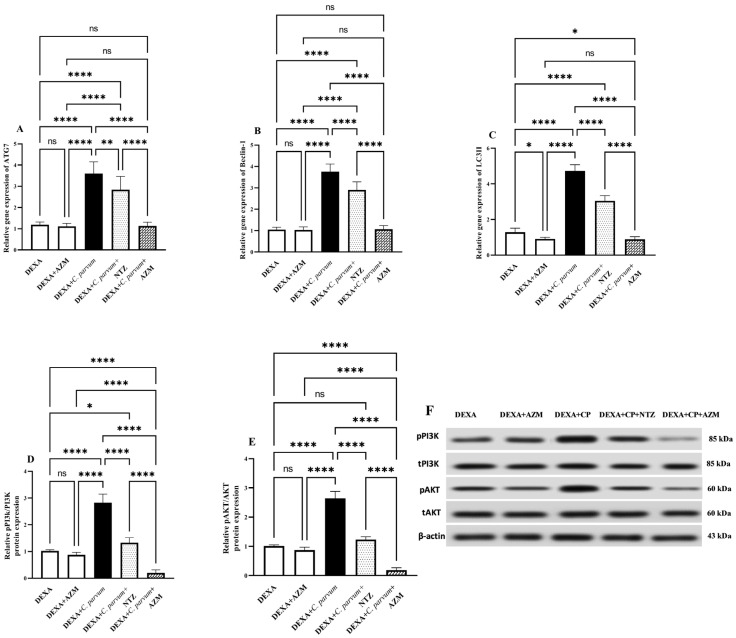
AZM inhibited the activated autophagic machinery in *C. parvum*-driven ileocecal adenocarcinoma. Relative expression of (**A**) ATG7, (**B**) Beclin-1, and (**C**) LC3II genes (*n* = 8). Relative expression of (**D**) pPI3K/PI3K and (**E**) pAKT/AKT proteins. (**F**) Representative Western blot picture for tPI3K, pPI3K, tAKT, and pAKT. Data are displayed as mean (*n* = 8) ± SD, * *p* < 0.05, ** *p* < 0.01, **** *p* < 0.0001, ns: non-significant. DEXA: dexamethasone; NTZ: nitazoxanide; AZM: azithromycin.

*Cryptosporidium* infection markedly induced autophagy in immunosuppressed mice compared to the DEXA-administered group, as demonstrated by upregulated gene expression of Beclin-1 and ATG7, autophagy initiation markers. This was associated with upregulation in LC3II mRNA expression, indicating increased autophagosome formation. NTZ administration partially declined mRNA expression of ATG7, Beclin-1, and LC3II compared to mice infected with *C. parvum*. Notably, AZM resulted in substantial autophagy suppression, as demonstrated by downregulated Beclin-1, ATG7, and LC3II genes expression relative to the *C. parvum*-infected or NTZ-treated groups. Moreover, AZM showed comparable autophagy inhibition to either DEXA or AZM + DEXA-treated groups (Figure 5A–C).

### 3.5. Azithromycin Inhibited the PI3K/AKT Signaling in the Ileocecal Adenocarcinoma Driven by Chronic Cryptosporidiosis

*C. Parvum*-infected mice considerably upregulated the protein expression levels of pPI3K and pAKT. NTZ administration moderately reduced the pPI3K and pAKT protein expressions when compared to the *C. parvum*-infected group, though they remained significantly elevated relative to the DEXA-treated group. AZM administration counteracted the upregulation in PI3K/AKT signaling compared to mice infected with *C. parvum* or treated with NTZ (Figure 5C–F).

### 3.6. Azithromycin Activated the Intrinsic-Induced Apoptosis in Ileocecal Adenocarcinoma Tissues Driven by Chronic Cryptosporidiosis

**Figure 6:**
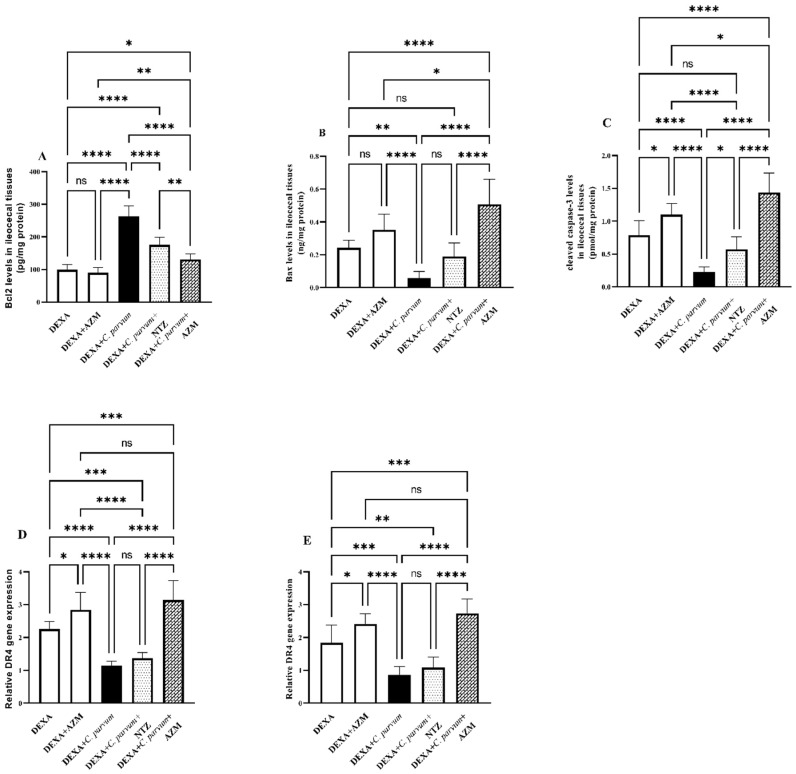
AZM triggered the intrinsic and TRAIL-mediated apoptotic pathways in *C. parvum*-driven ileocecal adenocarcinoma. Ileocecal levels of (**A**) Bcl2, (**B**) Bax, and (**C**) cleaved caspase-3. Relative gene expression of (**D**) DR4 and (**E**) DR5 in ileocecal tissues. Data are displayed as mean (*n* = 8) ± SD, * *p* < 0.05, ** *p* < 0.01, *** *p* < 0.001, **** *p* < 0.0001, ns: non-significant. DEXA: dexamethasone; NTZ: nitazoxanide; AZM: azithromycin.

Chronic infection with *C. parvum* was associated with a marked elevation in Bcl-2 levels and a marked reduction in Bax and cleaved caspase-3 relative to the DEXA-treated group, suggesting that persistent *Cryptosporidium* infection mitigates apoptosis. NTZ treatment demonstrated partial reduction in Bcl2 levels, associated with a moderate elevation in levels of Bax and cleaved caspase-3 compared to mice infected with *C. parvum*. Moreover, the AZM administration showed a prominent reduction in Bcl2 levels and an increase in the levels of Bax and cleaved caspase-3 relative to *C. parvum-infected* or NTZ-treated animals, as presented in Figure 6A–C.

### 3.7. Azithromycin Enhanced TRAIL-Induced Cell Death in Ileocecal Adenocarcinoma Tissues Driven by Chronic Cryptosporidiosis

*C. parvum*-infected mice suppressed the TRAIL-mediated apoptotic pathway, as denoted by a marked downregulation in DR4 and DR5 gene expression compared to mice treated with DEXA. Administration of NTZ did not significantly affect the TRAIL pathway compared to the group infected with *C. parvum*. However, the administration of AZM counteracted the *Cryptosporidium*-induced apoptosis inhibition, as demonstrated by a considerable upregulation in the mRNA expression of DR4 and DR5 relative to the *C. parvum*-infected or NTZ-treated groups (Figure 6D,E).

## 4. Discussion

Cryptosporidiosis remains a significant cause of diarrheal diseases in humans and animals, especially in immunocompromised hosts. Cryptosporidiosis management is still challenging owing to the limited efficacy of available therapeutics, such as NTZ. Additionally, the molecular mechanisms implicated in *C. parvum*-induced ileocecal adenocarcinoma are not fully elucidated. Hence, a deeper understanding of host–parasite interactions can decipher its pathogenesis and develop effective therapeutic strategies [55].

This study evaluated the efficacy of AZM, a macrolide antibiotic with antimicrobial and immunomodulatory properties, for the treatment of cryptosporidiosis and its associated ileocecal adenocarcinoma. Although some studies have questioned its parasitological efficacy against *Cryptosporidium* infections, an increasing body of clinical evidence and case reports supports its ability to reduce parasitic load and improve clinical symptoms. Building on the existing evidence and considering the limited therapeutic options, apart from NTZ, our study provides a deeper understanding of AZM’s therapeutic potential against chronic *C. parvum* infections. We also elaborated on its role in impeding the parasite-driven mechanisms that contribute to the development of ileocecal adenocarcinoma.

Our results demonstrated AZM’s ability to reduce parasitic burden by 87% compared with the *C. parvum*-infected group and to outperform NTZ treatment (62%). AZM was previously reported to reduce the parasitic burden by 100% in diarrheic calves [56]. In contrast, others reported that AZM alleviated diarrheal symptoms without eliminating oocyst excretion in cryptosporidiosis-infected piglets [57]. The variable outcomes after AZM usage as a therapeutic for cryptosporidiosis could be attributed to differences in regimens, including duration or route of administration.

Persistent *C. parvum* infection triggers pro-tumorigenic immune responses, allowing the parasite to evade the host’s immune defenses and thrive within IECs. The parasite exacerbates inflammatory reactions within IECs mediated by elevated pro-inflammatory cytokines, including IL-1β, TNF-α, and IL-6, along with chemokines recruiting immune cells to the infected tissues [3,58]. This results in an immunosuppressive tumor environment by enhancing cellular proliferation and autophagy, mitigating apoptosis, and promoting tumorigenic pathways [3]. In our study, chronic *C. parvum* infection triggered a pro-inflammatory microenvironment manifested by persistently elevated cytokines, including IL-6 and TNF-α, in the ileocecal tissues of infected mice. Our histopathological examinations also revealed extensive infiltration of the ileum and ileocecal tissues, mainly with mononuclear inflammatory cells.

IL-6 and TNF-α are recognized as promoters for cancer progression in *C. parvum* infections [3], where IL-6 is linked with tumor initiation and progression in response to sporozoites or soluble antigens [59,60]. Moreover, TNF-α, a key pro-inflammatory cytokine, is released by inflammatory monocytes, facilitating *C. parvum* infection by destroying the intestinal barrier. This leads to the propagation of infection and to tumor progression through upregulating cancer-associated genes in the epithelial cells, including those involved in extracellular matrix remodeling [61]. NTZ administration partially reduced their levels, whereas AZM counteracted the exacerbated inflammatory responses induced by persistent *C. parvum* infection. This could be attributed to AZM’s immunomodulatory and anti-inflammatory effects [62], dampening cancer progression driven by inflammation.

Autophagy is a conserved cellular-homeostasis-maintaining mechanism that serves as a host defense mechanism against invading pathogens [63]. However, dysregulated autophagy can contribute to cancer development by promoting cancer cell proliferation and tumor growth, often through the activation of signaling pathways such as the PI3K/AKT pathway [64]. However, *C. parvum* exploits autophagic machinery in host IECs to promote its survival, thereby creating a favorable environment for replication and contributing to ileocecal adenocarcinoma progression [3,65]. Previous in vitro studies documented that *C. parvum* controls the host autophagic machinery by hyperactivating the autophagy-dependent proteins [65,66,67] and the PI3K/AKT signaling pathway [65]. Consistent with these findings, our study demonstrated that chronic *C. parvum* infection substantially triggered autophagy in IECs, thereby contributing to cell survival and ileocecal adenocarcinoma progression. This was evidenced by the markedly upregulated expression of key autophagy-related proteins, including LC3II, Beclin 1, and ATG7, which are also implicated in tumor progression [64]. LC3II is the main indicator of an increase in the autophagic flux [68], while Beclin-1 and ATG7 are critical components of autophagosome nucleation and formation, respectively [69]. Moreover, *C. parvum* infection markedly upregulated PI3K and AKT protein expression, further relating *C. parvum*-induced autophagy to its tumorigenic potential [3]. NTZ partially suppressed autophagy-related proteins and PI3K/AKT activation.

In contrast, treating *C. parvum*-infected mice with AZM inhibited autophagy and suppressed the development of ileocecal adenocarcinoma. This is reflected by the pronounced suppression of LC3II, Beclin-1, and ATG7, which is associated with a marked downregulation of PI3k/Akt signaling. AZM’s activity on the PI3K/AKT signaling pathway has been previously reported in various diseases [29,70] and is found to be affected by the tissue and disease type. Although it activated PI3K/AKT to promote M2 macrophage polarization in systemic lupus erythematosus [70], our study demonstrates that AZM not only aids in parasite clearance but also mitigates tumor cell proliferation in *Cryptosporidium*-induced adenocarcinoma. These effects align with its previously reported inhibitory potential for PI3K/AKT and autophagy in cancer therapy [29,71].

Interestingly, apoptosis and autophagy function together as survival mechanisms for *C. parvum* infection. *C. parvum* manipulates the intricate interplay between these mechanisms during infectivity and in the pathogenesis of ileocecal adenocarcinoma [72]. Typically, autophagy acts in opposition to apoptotic pathways, both the intrinsic mitochondrial and extrinsic TRAIL-mediated pathways, in *C. parvum* infection [73,74] and cancer progression [75]. *C. parvum* infection induces apoptosis resistance in host IECs via elevating the levels of the Bcl2 anti-apoptotic protein, and reducing the pro-apoptotic protein “Bax” and cleaved caspase-3 [76]. In addition, it confers resistance to apoptosis via TRAIL-mediated apoptosis [67,77]. Apoptosis resistance supports the parasite’s survival and replication [14,78], as well as gastrointestinal cancer induction [79]. Likewise, our data showed that chronic *C. parvum* infection mitigated Bax and cleaved caspase-3 and elevated Bcl2 levels, indicating resistance to intrinsic apoptotic pathways. Bcl2 is reported to interact with Beclin-1, facilitating autophagy while suppressing mitochondrial apoptosis [62], which is consistent with our results. Additionally, *C. parvum* infection activates the PI3K/Akt pathway, mitigating caspase-mediated apoptosis and fostering a tumorigenic environment [3], findings that also parallel our data. Moreover, *C. parvum* infection significantly downregulated the TRAIL-mediated apoptotic pathway, as reflected by the reported downregulation in the death receptors “DR4 and DR5” mRNA expression in our study, consistent with previous reports [77]. These findings reinforce that *C. parvum*-induced suppression of both apoptotic pathways and the activation of autophagy enables the parasite’s persistence within the infected host IECs, facilitating replication and progression toward adenocarcinoma. NTZ treatment partially restored the intrinsic apoptotic pathway, as indicated by moderate increases in Bax and cleaved caspase-3 levels, and a reduction in Bcl2 levels. However, it showed no significant induction of TRAIL-mediated apoptosis, with effects comparable to those in mice infected with *C. parvum*. Notably, administration of AZM to mice infected with *C. parvum* significantly restored the balance between the intrinsic and death receptor-mediated apoptotic pathways in the ileocecal tissues. Moreover, AZM’s anticancer activities involved disrupting mitochondrial membrane potential, leading to cytochrome c release, Bax activation, and Bcl2 suppression [80,81]. Simultaneously, AZM upregulated the extrinsic apoptotic pathway by enhancing the expression of death receptors (DR4 and DR5) and activating downstream caspases, including caspase-3 [28]. Our findings were also coupled with modulated autophagy, thus counteracting the parasite’s survival strategies and facilitating the elimination of infected cells. By regulating the interconnected autophagy and apoptosis survival pathways, AZM overcomes apoptotic resistance, thereby inhibiting *C. parvum*-induced ileocecal adenocarcinoma.

Our study has some limitations. First, our findings are limited to a single *Cryptosporidium* species, *C. parvum*, which limits generalizability to other *Cryptosporidium* species or genetic variants. Second, while short-term AZM treatment potently inhibited PI3K/AKT signaling and autophagy in our model, emerging preclinical evidence suggests potential adverse effects with chronic AZM exposure, including inducing tumor proliferation in melanoma and fibrosarcoma models by sustaining autophagy inhibition [82] and cardiotoxicity when combined with narcotics like capatgon, via upregulation of PI3K/AKT/NF-κB signaling [83]. Such reported effects likely require dose-, duration-, and disease-context-dependency, and cardiac monitoring for at-risk patients receiving prolonged AZM treatment. Despite these limitations, our study provides evidence that AZM alleviates *C. parvum*-driven oncogenic signaling and warrants further investigation.

## 5. Conclusions

In this study, we provide, to the best of our knowledge, the first in vivo evidence that chronic *C. parvum* can promote ileocecal cancer progression by manipulating the critical balance between autophagy and apoptosis in the host’s IECs. Moreover, we demonstrate that AZM achieves superior parasite clearance compared to standard NTZ therapy while simultaneously suppressing chronic inflammatory responses and the pro-tumorigenic cellular environment. By inhibiting autophagy, attenuating inflammation, and reactivating both intrinsic and TRAIL-mediated apoptotic pathways, AZM effectively targets both the pathogen and the host mechanisms it exploits. Our findings highlight the dual therapeutic efficacy of AZM, a drug with an established clinical safety profile, in mitigating chronic cryptosporidiosis and its associated cancer risk, providing a robust foundation for developing improved therapeutic strategies against persistent *C. parvum* infections and their severe complications.

## Figures and Tables

**Table 1:** Oligonucleotide primers utilized in real-time PCR.

Gene	Forward (5′-3′)	Reverse (5′-3′)
D4 (*Tnfrsf10a*)	TTCTCAGCCACTCTGGCT	AGAGGATCTGAGAAATG
D5 (*Tnfrsf10b*)	AGTAACAGCTAACCCAG	GCAGAGAGGGTATTGAC
LC3 II (*Map1lc3b*)	TTGGTCAAGATCATCCGGCG	CTCTTCCAGGGCCGTGTAGA
Atg 7	CTGTACGATCCCTGTAACCTAACCC	CGAAAGCAGAGAACTTCAACAGCT
Beclin-1 (*Becn1*)	AGCACGCCATGTATAGCAAAGA	GGAAGAGGGAAAGGACAGCAT
GAPDH	GACGGCCGCATCTTCTTGA	CACACCGACCTTCACCATTTT

## Data Availability

The original contributions presented in this study are included in the article. Further inquiries can be directed to the corresponding author.

## Abbreviations

The following abbreviations are used in this manuscript:

AZM	Azithromycin
NTZ	Nitazoxanide
*C. parvum*	*Cryptosporidium parvum*
AIDS	Acquired immune deficiency syndrome
IECs	Intestinal epithelial cells
PI3K	Phosphoinositide 3 kinase
TRAIL	Tumor necrosis factor-related apoptosis-inducing ligand
DEXA	Dexamethasone
PCR	Polymerase chain reaction
